# Prevalence and factors associated with substance use among students in tertiary institutions in Buea, Cameroon

**DOI:** 10.11604/pamj.2022.41.103.29272

**Published:** 2022-02-04

**Authors:** Conrald Ekukole Metuge, Anastase Dzudie, Peter Vanes Ebasone, Jules Clement Nguedia Assob, Marcelin Ngowe Ngowe, Emmanuel Njang, Stephane Mabouna, Christian Eyoum

**Affiliations:** 1 Clinical Research Education, Networking and Consultancy, Yaoundé, Cameroon,; 2 Faculty of Medicine and Biomedical Sciences University of Yaoundé 1, Yaoundé, Cameroon,; 3 Service of Internal Medicine and Subspecialties, Douala General Hospital, Douala, Cameroon,; 4 Department of Medical Laboratory Sciences, Faculty of Health Sciences, University of Buea, Buea, Cameroon,; 5 Department of Surgery and Specialties, Faculty of Medicine and Pharmaceutical Sciences, University of Douala, Douala, Cameroon,; 6 Health Education and Research Organisation, Yaoundé, Cameroon,; 7 Service of Psychiatry, Laquintinie Hospital, Douala, Cameroon

**Keywords:** Prevalence, substance use, students, tertiary institutions, associated factors

## Abstract

**Introduction:**

substance use is a major global public health problem. About 5.6% of the global population aged 15-64 years consumed a drug at least once in 2016. The use of substances by youth, especially students in tertiary schools is increasing rapidly worldwide. This rise in substance use is associated with a negative impact on student's health, as well as their professional and social life.

**Methods:**

in a cross-sectional institution-based survey we recruited 650 students by convenience sampling from 3 randomly selected tertiary institutions within the Buea municipality. Data was collected using a pre-tested self-administered World Health Organization (WHO) model core questionnaire to collect information on sociodemographic data and use of various substances. The data collected were entered into Microsoft Excel 2016 and exported to SPSS version 24. Descriptive analysis was done to examine findings, and multivariate logistic regression models were used to determine factors independently associated with substance use.

**Results:**

of the 650 students recruited, 625 consented and completed the questionnaire, for which 67.4% were females with a mean age of 22.2 ± 2.837 years. The overall prevalence of substance use was 89.9%. The prevalence of multiple substance use was 29.9%. The most currently used substances were tobacco (26.2%), alcohol (19.7%), tramadol (2.8%) and cannabis (2.0%). The main reason for substance use was to relieve stress (relax) 91.7%. The main negative effects reported were quarrel or arguments (18%) and loss of money (16.7%). Peers (66.9%) were the prime source of substance use. On multivariate analysis, male sex was the principal predictor for substance use (95% CI): 0.801 (1.128, 4.398).

**Conclusion:**

the prevalence of substance use is high among students in tertiary institutions in Buea. Multilevel, value-based, comprehensive, and strategic long-term intervention plans are required to curb this problem.

## Introduction

Psychoactive substances are believed to provide pleasure because they give inner peace and satisfaction, alter perception and heighten sensation [1]. Despite its pleasurable effects, substance use has negative effects on the health, family, social, and professional life of people, making it a major public health concern [2]. In 2017, an estimated 271 million people of the global population, aged 15-64, had used drugs in the previous year. This is 30% higher than the 210 million reported in 2009 [3]. This has a high impact in terms of health consequences. The global disease burden attributable to alcohol and illicit drugs is estimated at 5.4%, while 3.7% is attributable to tobacco use alone [4]. Globally, 585,000 people are estimated to have died as a result of drug use in 2017. The most consumed psychoactive substance globally is alcohol. However, the preferred illicit substance consumed in Africa is cannabis, while opiates are the most consumed illicit substance in Europe and Asia and cocaine predominating in South America [4].

Substance use and misuse is on a gradual rise worldwide and most especially in low and middle-income countries where policies on substance use are still quite rudimentary [5-7]. Several studies have indicated that substance use is common among students and is becoming increasingly widespread in many African countries [8-10]. The rapid change in socio-economic and cultural aspects plus Westernization of most countries in sub-Saharan Africa is creating a favorable ground for substance use [5,11]. In Cameroon, the national anti-drugs committee reports that 21% of the population had used substances, 60% of whom are youths aged 20-25 years (some of whom are students in tertiary schools). Moreover, more than, 12000 young people less than 15 years of age use narcotic drugs and other psychotropic substances [12].

Life in tertiary education is a period when students transition to independence and freedom from direct supervision of their families, self-decision-making, and intense academic pressure. They share living areas with strangers, form new social groups and friends, balance social engagements with academic and other life responsibilities. These expose them to certain social norms which youths uphold, which are divergent from the values they originally adopted from their parents. These perceived norms motivate the youth to indulge in unhealthy behaviors such as smoking and alcohol and drug use [5,8,13,14]. Some common substances used by students in tertiary institutions are: tobacco, alcohol and opioids [5,6,8]. Alcohol and tobacco are seen as ''gateway'' substances´ as per the gateway drug theory; which suggests that using soft substances like cannabis, alcohol and tobacco is a behavioral doorway that leads to consumption of the so-called hard drugs like heroin, cocaine, crystal methamphetamine and ecstasy [15]. In Cameroon, alcohol aside, the most common substance in demand is cannabis (58.54%) usually associated with tobacco. Other consumed substances in Cameroon include: tramadol (44.62%), cocaine (12.10%), traditional makeshift preparations (7.59%), solvents (7.36%) and heroin (5.7%) [12]. Mbanga *et al*. reported tramadol and cannabis as the most consumed recreational substances among medical and nursing students in Cameroon [16]. Several studies have shown that these substances have severe negative effects such as poor grades at school, violent acts (arguments, fights), poor sleep quality, robbery, accidental injuries, engagement in unprotected sex, dependence/addiction and problems with their teachers, friends and parents as well as fuelling insurgency and terrorism [6,13]. There is a deficit of data on substance use in Cameroon and more specifically among university students. This has a serious effect on any attempt through interventions to solve this ever-growing problem. Therefore, the thrust of this study is to assess the magnitude, pattern and factors associated with substance use among students in tertiary institutions in Buea, Cameroon.

**Objectives:** to study the epidemiology of substance use among students in three tertiary institutions in Buea. By assessing the prevalence of substance use, the commonest substances used, the negative effects of these substances and the factors associated with substance use on these students. To assess the prevalence of substance use among students from three tertiary institutions in three tertiary institutions in Buea. To identify the substances commonly used by students in three tertiary institutions in Buea. To identify the negative effects of these substances on students in three tertiary institutions in Buea. To identify the factors associated with substance use among students in three tertiary institutions in Buea.

## Methods

**Study design:** this was an institution based descriptive cross-sectional study.

**Study area and period:** the study took place over a period of 4 months (January 27^th^, 2019 to April 15^th^, 2019) at three tertiary institutions in Buea (University of Buea (UB), Catholic University Institute of Buea (CUIB) and Higher Institute of Management Studies Buea (HIMS)). Buea is the capital of the South West Region. It is located on the eastern slopes of Mount Cameroon, West of the Mungo, near the coastal city of Limbe at the shore of the Atlantic Ocean in the Fako division. The University of Buea is a state-owned university with a student population of over 18000 comprising undergraduate and post graduate students and about 5>00 teaching staff. It consists of 10 faculties and schools combined. It consists 4 campuses (main campus, Faculty of Health Sciences campus and the College of Technology and Higher Technical Teachers´ Training College in Kumba). Only the main campus was included in the study. The Catholic University Institute of Buea (CUIB) is a private university in Buea. Catholic university institute of Buea has a student population of 1204 undergraduate students and 120 staff. The university is made of 6 schools/colleges. It has 2 campuses (Buea and Douala campus). Only the Buea campus was included in the study. The Higher Institute of Management Studies (HIMS) Buea is a private Higher Education Institution with the express aim of grooming professionally qualified managers for modern industries. It offers a two-year program at the end of which students receive a higher national diploma.

**Selection criteria:** only students who were 18 years and above from the selected tertiary institutions irrespective of their year of study were enrolled in our study. Excluded from the study were all who did not consent, were seriously ill and absent during the study period.

**Sampling technique:** the three tertiary institutions were first randomly selected from the 25 approved in Buea by the Ministry of Higher Education in Cameroon. Students were then selected from each institution via convenience sampling.

### Operational definitions variables

**Substance:** chemical substance that acts primarily on the central nervous system where it alters brain function, resulting in temporary changes in perception, mood, consciousness and behaviour.

**Substance use:** substance use is defined as use of psychoactive substances such as alcohol, cigarette and other drugs for non-medical and non-scientific purposes which results in alteration of mood, behavior, perception and consciousness.

**Current user:** a person who consumed any substance at least once in the past 30 days.

**Ever user:** referred to as use of any of the substances at least once in an individual´s lifetime.

**Data collection tool and procedure:** a self-administered questionnaire from the Global Assessment Program on Drug Abuse (GAP) (Toolkit Model 3) [17] which was adopted from the original WHO model core questionnaire for youth was used to collect information on the use of various substances including alcohol, tobacco, stimulants, marijuana, cocaine and heroin, among others as well as demographic and social data of each student. The sample population was recruited from students on campus. Potential participants were informed on the aim and objectives of the study. Before obtaining written informed consent, the ethical principles were discussed with the students which involved voluntary participation in the study, anonymity of the participants, confidentiality of the data and their right to withdraw from the study at any point in time with no negative consequences.

**Data quality assurance:** the questionnaire was pretested on 20 students of a nearby university not included in the study population. The data collectors (facilitators) were trained over 2 days, and proper instructions were given before the survey. The collected data were reviewed and checked for completeness before data entry.

**Sample size determination:** the sample size was calculated using the formula:


$$n=\frac{z^{2}p(1-p)}{d^{2}}$$

Where n is minimum sample size; Z is standard normal variation which will be set at 1.96 (p value: 0.05 and 95% confidence interval); p is proportion in the target population. That is, the prevalence of substance use in university students according to a study done in Sudan which was at 31%. Therefore, we assume p=31% equivalent to 0.31 and d =the target margin of error put at 0.05 (for a 95% confidence interval). The calculated sample size was 329. Upon considering a potential non-response of 10%, the sample size was increased to 362. To conduct a meaningful analysis, the final sample was raised to 650.

**Data analysis:** all data from each participant´s questionnaire were coded and entered into Microsoft Excel on a computer. Questionnaires were kept in a safe place accessible to the principal investigator only. Data cleaning was done by carrying out range and consistency checks. Descriptive and analytical statistics was carried out using IBM SPSS version 24. Descriptive statistics was done to describe the study population. Bivariate and multivariate analyses were employed to identify factors associated with the outcome variable. Odds ratio with 95% confidence interval was computed to assess the level of association and statistical significance. Those variables which were found to be significant in the bivariate analysis were retained for further multivariate analysis. Then logistic regression analysis was done to control confounding variables and to predict independent factors associated with substance use. A p-value of ≤ 0.05 was considered statistically significant and the confidence interval was set at 95%.

**Ethical considerations:** after obtaining ethical approval (application number: 896-01) from the Institutional Review Board (IRB) of the Faculty of Health Sciences (reference: 2019/896-01/UB/SG/IRB/FHS), University of Buea, authorisation from the University of Buea, Higher Institute of Management Studies and Catholic University Institute of Buea were then obtained. Students were visited in their classes and oriented on the survey and questionnaire. All recruited students provided signed informed consent. Students were assured that information would be maintained confidential. The questionnaire did not collect any information that could be used to identify the students.

## Results

**Socio-demographic characteristics:** six hundred and fifty (650) students were enrolled in this study with 625 consenting to participate giving a response rate of 96.1%. Majority were female; (67.4%) with a female to male ratio of 2: 1. The mean age of the respondents was 22.2 ± 2.837 years with majority of them (62.7%) being within the 21-25 years age bracket. Almost all the respondents (98.1%) were of the Christian faith and 43.6% of the students lived with parents or guardians. Majority of the study participants were in second (29.8%) and third (32.6%) year of study. The median monthly allowance was 27000FCFA ($48.46) with an inter-quartile range of 15000-40000FCFA ($27-$72) (Table 1).

**Table 1:** socio-demographic characteristics of the respondents (N=625)

Variable	Male (n=204) n (%)	Female (n=204) n (%)	Total (n=625) n (%)
**Age groups (in years)**			
<20	38 (18.6)	134 (31.8)	172 (27.5)
21 - 25	136 (66.7)	256 (60.8)	392 (62.7)
26 - 30	29 (14.2)	26 (6.2)	55 (8.8)
31 - 35	1 (0.5)	3 (0.7)	4 (0.6)
>35	0	2 (0.5)	2 (0.3)
**Religion**			
Christian	199 (97.6)	414 (98.3)	613 (98.08)
Muslim	5 (2.4)	7 (1.7)	12 (1.92)
Others	0 (0.0)	0 (0.0)	0 (0.0)
**Living conditions**			
Parents/guardians	78 (28.2)	195 (46.3)	273 (43.6)
Alone	82 (40.2)	158 (37.5)	240 (38.4)
Friend/peer	40 (19.6)	51 (12.1)	91 (14.6)
Other	4 (2.0)	17 (4.03)	21 (3.4)
**Marital status**			
Married	1 (o.5)	16 (3.8)	17 (2.7)
Divorced/separated	1 (0.5)	1 (0.2)	2 (0.3)
Living as a couple	5 (2.5)	10 (2.4)	15 (2.4)
Never married/single	197 (96.5)	394 (93.6)	591 (94.6)
**Year of study**			
First year	54 (26.5)	91 (21.6)	145 (23.2)
Second year	42 (20.6)	145 (34.4)	187 (29.8)
Third year	65 (31.9)	139 (33.0)	204 (32.6)
Fourth year or higher	43 (21.1)	46 (10.9)	89 (14.2)
**Source of income**			
None	0 (0.0)	1 (0.2)	1 (0.1)
Salary/ wages from a job	33 (12.8)	16 (3.3)	49 (6.5)
Welfare, government assistance	1 (0.4)	4 (0.7)	5 (0.7)
Family or spouse	188 (72.9)	409 (83.1)	597 (79.6)
Friends	29 (11.2)	61 (12.4)	90 (12)
Illegal income	3 (1.2)	0 (0.0)	3 (0.4)
Other	4 (1.6)	1 (0.2)	5 (0.7)

** ; multiple responses

**Figure 1:**
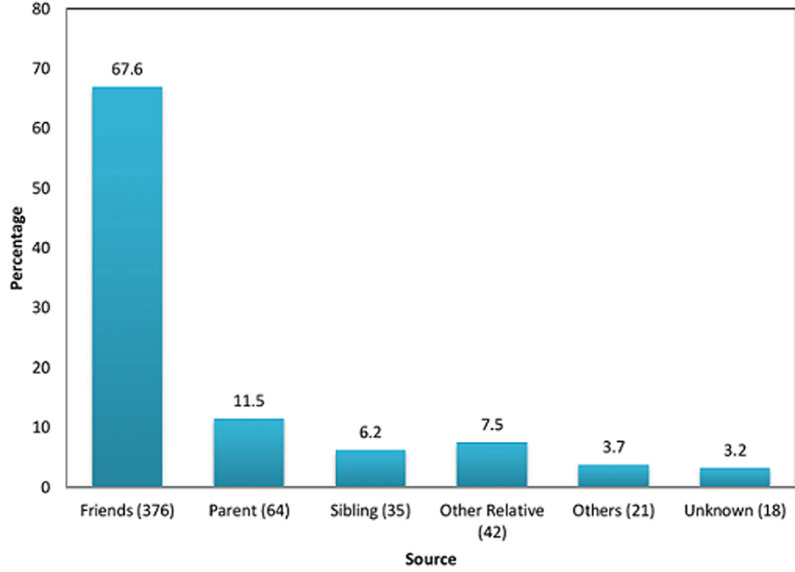
sources of substances used

**Magnitude of substance use:** the overall prevalence of substance use was 89.9%. Single substance users constituted 70.1% and multiple substance users 29.89%. The life time prevalence of substance use was 89.9%, the overall past year prevalence of substance use was 77% and the overall current prevalence was 61.6%. The lifetime, past year, and past month prevalence of substance use is displayed in Table 2. Lifetime use of alcohol was reported by 553 (98.5%) students, followed by tobacco (28.3%), tramadol (7.5%), and cannabis (6.8%). A lesser proportion of students reported lifetime use of other opiates (1.1%), drugs by injection (0.8%), solvent inhalants (0.7%), cocaine (0.7%) and heroin (0.4%). There was no reported use of amphetamines, lysergic acid diethylamide and ecstasy. The commonest reasons for consuming these substances include: to relieve stress (91.7%), curiosity (35.8%) and peer pressure (13.7%). Other (1.8%) reasons included depression, for chronic pain, they are cheap, for sexual enhancement, to be courageous, to help them sleep (Table 3). Of the negative effects of substance use (Table 3), quarrel/argument (18%), loss of money or other valuable items (16.7%) and problems in relationships with parents and friends (12.3%) were the commonest. They also reported other negative effects such as engagement in unprotected sexual intercourse and poor performance at school. The main sources of substance use were friends (66.9%). Other sources include parent, sibling or other relatives. In 3.2%, the source was undocumented (Figure 1).

**Table 2:** lifetime, past year and current prevalence of substance use (n=562)

Drug/ substance	Lifetime use	Last 12 months use	Current user
Alcohol	553 (98.2)	475 (84.5)	376 (66.9)
Tobacco	159 (28.3)	150 (26.8)	147 (26.2)
Tramadol	42 (7.5)	28 (5.0)	16 (2.8)
Cannabis	38 (6.8)	21 (3.7)	11 (2.0)
Otheropiates	6 (1.1)	3 (0.5)	0 (0.0)
Drugs by injection	5 (0.8)	4 (0.6)	4 (0.6)
Cocaine	4 (0.7)	2 (0.4)	1 (0.2)
Heroin	2 (0.4)	2 (0.4)	2 (0.4)
Solvents	4 (0.7)	2 (0.4)	1 (0.2)
Lysergic acid diethylamide	0 (0.0)	0 (0.0)	0 (0.0)
Amphetamines	0 (0.0)	0 (0.0)	0 (0.0)
Ectasy	0 (0.0)	0 (0.0)	0 (0.0)

**Table 3:** reasons for consuming substances and negative effects (n=502)

Reason	Frequency	Percentage
Relieve stress	515	91.7
Curiosity	201	35.8
Peer pressure	77	13.7
Cope with problems	52	9.3
Easily available	47	8.4
Excess pocket money	27	4.8
Enhance academic performance	18	3.2
Others	10	1.8
No reason	7	1.2
**Negative effects**		
Quarrel or argument	101	18.0
Loss of money or other valuable items	94	16.7
Problems in your relationship with your parents	69	12.3
Problems in your relationship with your friends	69	12.3
Damage to objects or clothing	66	11.7
Engaged in unprotected sex	63	11.2
Engaged in sex you regretted the next day	49	8.7
Performed poorly at school or work	49	8.7
Scuffle or fight	39	6.9
Victimized by robbery	35	6.2
Accident or injury	33	5.9
Trouble with police	26	4.6
Health problems	24	4.3
Problems in your relationship with your teachers	11	2.0

**Factors associated with substance use:** bivariate analysis was done for the following socio-demographic variables: age, gender, religion, living conditions, marital status, year of study and median monthly allowance. Being male was associated with substance use (p=0.001). Living with parents or guardians was significantly associated with less substance use (p=0.053) as well as having a median monthly allowance of less than 27000FCFA (0.029). Comparisons between different segments of socio-demographic factors and current (last 30 days) substance use are displayed on Table 4. After controlling for the effects of potentially confounding variables using multivariate logistic regression model, male gender (p=0.02) was independently associated with substance use.

**Table 4:** factors associated with current (past 30 days) substance use (bivariate analysis) (n=385)

Variable	Yes (%)	No (%)	Odds ratio	Confidence interval	p-value
**Age**					
15 - 25 years	323(63.1)	189(36.9)	1.406	0.931-2.122	0.129
25 years or more	62(54.9)	51(45.1)			
**Gender**					
Male	146(71.6)	58(28.4)	1.917	1.337-2.748	**0.001**
Female	239(56.8)	182(43.2)			
**Religion**					
Christian	377(61.5)	236(38.5)	0.799	0.238-2.682	1.000
Muslim	8(66.7)	4(33.3)			
**Living condition**					
With Parents/ Guardians	156(57.1)	117(42.9)	0.716	0.518-0.991	0.053
Alone or friends/ others	229(65.1)	123(34.9)			
**Marital status**					
Married/ living as a couple	17(53.1)	15(46.9)	0.693	0.339-1.415	0.409
Single/ divorced	368(62.1)	225(37.9)			
**Year of study**					
Year 1 - 2	193(58.1)	139(41.9)	0.730	0.528-1.011	0.070
Year 3 or higher	192(65.5)	101(34.5)			
**Median monthly - allowance**					
<27000FCFA ($46.56)	175(66.8)	87(33.2)	0.682	0.490-0.950	**0.029**
**>**27000FCFA ($46.56)	210(57.9)	153(42.1)			

## Discussion

We observed an overall prevalence of substance use of 89.9% majority of who consumed just a single substance (70.1%). Most of the participants were female, Christians and in their third year of tertiary educaion. The commonest substances used were alcohol (98.4%), tobacco (28.3%), tramadol (7.5%) and cannabis (6.8%). We also found out that some of the main reasons for consumption of substances were to have fun (65.7%), out of curiosity (35.8%), to relieve stress (26%), and peer pressure (13.7%). Moreover, we demonstrated that some of the main negative effects of substance use were quarrel/argument (18%), loss of money or other items (16.7%), problems in relationships with parents (12.3%). Friends (67.6%) were the greatest source of substances used. The male gender (p=0.02) was the only factor associated with substance use.

**Interpretation and generalisability:** the mean age of respondents in this study was 22.2 years (SD 2.837). This is similar to a study carried out in Cameroon by Mbanga *et al*. [16] on medical and nursing students. Duru *et al*. [9] in Nigeria and Gebrelassie *et al*. [14] in Ethiopia reported similar mean ages at 22.2 years (SD 3.8) and 22.2 years (SD 2.2) respectively. This study reported a higher proportion of female respondents. Similar studies have reported a preponderance of females in Cameroon and Sudan institutions of higher learning [13,16]. This is expected as most students at these institutions are females. However, other studies in sub-Saharan Africa had a male preponderance [5,6,9,14]. In line with the recent Sustainable Development Goals (SDGs), Cameroon like most African countries is gradually wiping out the gender gap in education. The lifetime prevalence rate of any psychoactive substance use among the respondents was 89.9%. This is higher than the 1.64% reported by Mbanga *et al*. [16] in Cameroon, 31% reported by Osman *et al*. [13] in Sudan, 45.3% reported by Duru *et al*. [9] in Nigeria, and 69.8% by Atwoli *et al*. [6] in Kenya. These differences could be as a result of the different criteria used for diagnosis of psychoactive substance use and the type of substance studied “such as alcohol which is frequently consumed by students and is relatively socially acceptable in different socio-cultural environments was not assessed by Mbanga *et al*. and Duru *et al*. but was assessed in this study.” The prevalence rate of psychoactive substance use within the past 12 months in this study was 77%. This was lower than the 45.5% reported by a similar study in South Western Nigeria [18]. This difference could be because alcohol was part of the psychoactive substance we studied unlike in thier study.

We observed that 29.8% of the students who had used multiple substances. However, this is higher than the 11.2% observed by Gebremariam *et al*. [10] in Ethiopia. This difference could be because the students in our study were probably less aware of the negative public health implications of these substances. Furthermore, the 'high' the students consuming multiple substances hoped to attain could not be provided by a single substance thus pushing them to consume multiple substances. On bivariate analysis of our data, living with parents or guardians was associated with less substance use. Similar studies reported this association in Sudan [13] and Nigeria [9]. This may stem from the fact that many students in higher education live with their families and those from different towns live with their relatives or guardians. This highlights the protective role of family and close relatives from negative peer influences on substance use. We also found that having an average monthly allowance lower than 27000FCFA ($48.46) was also associated with less substance use. This finding was similar to what Gebremariam *et al*. observed in Ethiopia [10]. This association could be explained by the fact that students who have higher monthly allowance will find it easier to purchase these substances. With the higher pocket allowance students will be tempted to buy these substances because they may have excess after spending on their school needs. Male gender was found to be independently associated to substance use. This finding was similar to the factors reported by Osman *et al*. [13], Tesfaye *et al*. [5] and Duru *et al*. [9] in Sudan, Ethiopia and Nigeria respectively. Male students are more exposed to these substances and peer pressure is more common in males than among female students. Moreover, many of the substances such as tobacco and alcohol are more socially acceptable if practiced among males.

**Limitation:** the main limitations of this study were the convenience sampling technique adopted in recruiting the study participants; and also, the fact that the data was self-reported, which could likely lead to bias. A longitudinal design in which students are followed up from admission to graduation would be most ideal. Also, the study may not be generalized to the entire youth population, as it involved only students in tertiary institutions and was a non-probabilistic study design.

## Conclusion

This study has demonstrated a high prevalence of substance use among students in tertiary institutions in a low-income country. With the main risk factor of substance use being the male gender. Thus, we propose goal directed programs to reduce and control the prevalence of substance use, raise students’ awareness and increase students' information about substance use disadvantages, especially in their enrollment in university. Students using substances should be provided with counselling and assisted with treatment options. The relevant legislative bodies should reinforce the measures or create new ones to reduce the ease with which students and the general population at large have access to psychoactive substances. The circumstances and factors associated with substance use differ according to the higher institutes. Thus, continuous research in other institutions in different towns in the country will help in monitoring trends and installing adequate preventive strategies.

### What is known about this topic

- Substance use is a major global public health issue and its prevalence is rapidly increasing in sub-Saharan Africa;
- Among the youth, substance use is a worldwide epidemic that can impact negatively on health, family, society, and educational and professional life.

### What this study adds

- It provides information on the prevalence of substance use in another low and middle-income country (Cameroon) among students in higher education; where there is an obvious paucity of such data;
- Moreover, the factors identified with and reasons stated for substance use in this study could be used to develop the most appropriate public health policies and preventive measures to help improve the health of this population;
- This study supported the notion that substance use is a problem which requires a joint effort by all the stakeholders involved in youth development to fully address the problem.

## References

[1] Adekeye OA, Adeusi SO, Chenube O, Ahmadu FO, Sholarin MA (2015). Assessment of alcohol and substance use among undergraduates in selected private universities in Southwest Nigeria. IOSR-JHSS.

[2] Schulte MT, Hser YI (2013). Substance use and associated health conditions throughout the lifespan. Public Health Rev.

[3] United Nations office on drugs and crime (2019). World drug report 2019.

[4] World Health Organization (2010). Atlas on substance use (2010): resources for the prevention and treatment of substance abuse disorders.

[5] Gezahegn T, Andualem D, Mitiku TH (2014). Substance use and associated factors among university students in Ethiopia: a cross-sectional study. J Addict.

[6] Atwoli L, Mungla PA, Ndung'u MN, Kinoti KC, Ogot EM (2011). Prevalence of substance use among college students in Eldoret, Western Kenya. BMC Psychiatry.

[7] Heydari ST, Izedi S, Sarikhani Y, Kalani N, Akbary A, Miri A (2015). The prevalence of substance use and associated risk factors among university students in the city of Jahrom, Southern Iran. Int J High Risk Behav Addict.

[8] Makanjuola AB, Daramola TO, Obembe AO (2007). Psychoactive substance use among medical students in a Nigerian university. World Psychiatry.

[9] Duru CB, Oluoha UR, Okafor CC, Diwe KC, Iwu AC, Aguocha CM (2017). Socio-demographic determinants of psychoactive substance use among students of tertiary institutions in Imo State, Nigeria. J Addict Res Ther.

[10] Gebremariam TB, Mruts KB, Neway TK (2018). Substance use and associated factors among Debre Berhan University students, Central Ethiopia. Subst Abuse Treat Prev Policy.

[11] Adeyemo FO, Beatrice O, Okpala PU, Oghale O (2016). Prevalence of drug abuse amongst university students in Benin City, Nigeria. Public Health Res.

[12] Bakary IT (2018). Culture, commercialisation et consommation des drogues: le plan de lutte du gouvernement. Cameroon Tribune.

[13] Osman T, Victor C, Abdulmoneim A, Mohammed H, Abdalla F, Ahmed A (2016). Epidemiology of substance use among university students in Sudan. J Addict.

[14] Gebreslassie M, Feleke A, Melese T (2013). Psychoactive substances use and associated factors among Axum university students, Axum Town, North Ethiopia. BMC Public Health.

[15] Vanyukov MM, Tarter RE, Kirillova GP, Kirisci L, Reynolds MD, Kreek MJ (2012). Common liability to addiction and “gateway hypothesis”: theoretical, empirical and evolutionary perspective. Drug Alcohol Depend.

[16] Mbanga CM, Efie DT, Aroke D, Njim T (2018). Prevalence and predictors of recreational drug use among medical and nursing students in Cameroon: a cross sectional analysis. BMC Res Notes.

[17] Global Assessment Programme on Drug Abuse (GAP) (2003). Conducting school surveys on drug abuse. United Nations Office on Drug and Crime.

[18] Onofa L, Taiwo A, Maroh I, Mofoluwake M (2016). Prevalence and pattern of drug abuse among students of tertiary institution students in Abeokuta, Ogun state, Nigeria. Int J Psychiatry.

